# Simultaneous umbilical blood flow during normal pregnancy in sheep and goat foetuses using non-invasive colour Doppler ultrasound

**DOI:** 10.21451/1984-3143-AR2017-976

**Published:** 2018-08-16

**Authors:** Mohammed Ahmed Elmetwally, Sabine Meinecke-Tillmann

**Affiliations:** 1 Institute of Reproductive Biology, University of Veterinary Medicine Hannover, 30559 Hannover, Germany.; 2 Faculty of Veterinary Medicine, Department of Theriogenology, Mansoura University, Mansoura 35516, Egypt.

**Keywords:** colour Doppler sonography, foetus, goats, umbilical, sheep

## Abstract

The characteristics of umbilical blood flow (UM) was investigated using 18 (25 foetuses) pregnant ewes and 20 (41 foetus) pregnant goats transrectal non-invasive color Doppler ultrasonographic examinations were done frequently between 2 and 8 week after breeding and then transabdominally until parturition. Colour Doppler velocimetery includes blood flow volume (BFV), time averaged maximum velocity (TAMV), resistance index (RI), pulsatility index (PI), time average of mean (TAMEAN) and impedance of blood flow (PS/ED or AB ratio). Also a qualitative evaluation of UM blood flow indicating increases (P < 0.001) in BFV, TAMV and TAMEAN were observed until 19 week of pregnancy in foetuses of sheep and goats and then those values decreased (P < 0.001) from 19 week until parturition. Conversely, UM-PI, RI and PS/ED decreased (P < 0.002-0.01) until 19 week and then increased (P < 0.01-0.0001). The umbilical artery BFV increased (P < 0.0001) during pregnancy from 7.27 ± 0.82 ml/min in sheep vs. 4.96 ± 0.54 ml/min in goats at 6 week of gestation to 700.51 ± 31.05 ml/min (~100 fold) in sheep *vs*. 665.56 ± 48.22 ml/min (~133 fold) in goats at 19 week and then decreased (P < 0.0001) to 350.561 ± 72.15 ml/min in sheep vs. 215.17 ± 35.06 ml/min in goats at 20 week. The absence of end diastolic velocity (EDV) of umbilical artery blood flow was detected in both species between 4 and 12 week of pregnancy. Results of this study clearly show that the non-invasive colour Doppler sonography can be used successfully to assess umbilical blood flow in foetuses of pregnant sheep and goats. These may provide guidelines for assessing the state of intrauterine fetal growth retardation in pregnancies of sheep and goats.

## Introduction

The first measures of umbilical blood flow were with an invasive procedure in which the lamb was exteriorized ( Cohnstein and Zuntz, 1884 ). The next trials involved exteriorization of the foetus for open chest measurements of cardiac output using a cardiometer (a tambour that measured stroke output; Barcroft *et al*., 1934 ) or use of chlorazol sky blue FF to regularly evaluate fetal blood volume during pregnancy ( Barcroft *et al*., 1939 ). An electromagnetic flowmeter technique was developed to measure the effect of quantitative reduction in umbilical blood flow in lambs under normal physiological conditions ( Dawes, 1968 ). Moreover, antipyrine was used to measure umbilical blood flow based on the Fick principle (antipyrine method; Rudolph and Heymann, 1967 , Crenshaw *et al*., 1968 ). The antipyrine was infused into veins in the hind limb of lambs and umbilical blood flow was then calculated after equilibration by the Fick method. These obtained values were comparable to those recorded simultaneously with electromagnetic flow meters. Then radioactive microspheres were used simultaneously to measure intrauterine umbilical blood flow in sheep foetuses ( Makowski *et al*., 1968 ). The first successful noninvasive transcutaneous continuous and pulsed wave Doppler evaluation of fetal umbilical arteries was performed 40 years ago ( Fitzgerald and Drumm, 1977 ; McCallum *et al*., 1978 ) in human. This method was used safely between 12 and 40 weeks of pregnancy in women.

Studies from Cohnstein and Zuntz (1884) until Makowski *et al*. (1968) were concerned principally with measuring the volume of blood flow using the antipyrine method, the blue dye method or the electromagnetic microspheres method before development of Doppler indices. Following this period and from the 1970s ( Soma *et al*., 1971 ; Anderson *et al*., 1977 ) and 1980s ( Trudinger *et al*., 1985 ) researchers were dependant on invasive Doppler measurements of umbilical blood flow especially in lambs. Recently, the noninvasive colour Doppler ultrasound was used to study uterine blood flow during pregnancy ( Elmetwally *et al*., 2016 ) as well as early puerperium (Elmetwally and Bollwein, 2017) in sheep and goats.

Considering the Doppler indices from umbilical blood flow studies, pulsatility index (PI), resistance index (RI), ( Hoskins *et al*., 1989 ) Vmax (maximum velocity of blood flow), and peak systolic/end diastolic (PS/ED; (AB ratio; Stuart *et al*., 1980 ; Elmetwally, 2016 ) are the most important.

Decreasing the AB ratio as the pregnancy advances ( Stuart *et al*., 1980 ) indicates decreasing vascular impedance and increasing vascular perfusion to the foetus. In pregnant goats, during all examinations of the umbilical artery in singleton and multiple pregnancies, there was no record of reverses in end-diastolic flow and no significant difference in PI and RI in single and multiple pregnancies ( Serin *et al*., 2010 ). The umbilical blood flow in human foetuses is characterized by an absence of end-diastolic velocity (EDV) between 8 and 12 week of gestation ( Coppens *et al*., 1996 ). The appearance of EDV is associated with a pronounced decrease in PI. Studies aiming to evaluate umbilical blood flow regularly throughout gestation in small ruminants are rare. Therefore, the present experiment evaluated qualitative and quantitative colour Doppler characteristics of umbilical blood flow throughout the gestation in foetuses of German Merino ewes and German improved Fawn goats.

## Materials and Methods

### Experimental animals

This study was conducted in accordance with German legislation on animal rights and welfare at the Institute for Reproductive Biology, University of Veterinary Medicine, Hannover, Germany during the natural breeding season (2008-2011). Eighteen pregnant German Merino ewes and 20 pregnant German improved Fawn goats were used in this study. The sheep were 5.5 ± 2.4 year of age (range 3-10 year) and the goats were 7 ± 1.9 year of age (range 2-10 year). The females were mated with males of proven fertility after synchronization of estrus using prostaglandin F2α (PGF). The day of onset of estrus was designated day 0 (day 0) of gestation in ewes and the day after onset of estrus was designated day 0 of pregnancy in goats.

Transrectal B mode ultrasound (7.5 MHz, Hitachi EUB 405, Hitachi Medical System, Japan) was used to confirm pregnancy in experimental animals. Doppler ultrasound examinations for experimental sheep and goats were done according to Elmetwally *et al*. (2016) . In brief, transrectal non-invasive colour Doppler (6 MHZ, Logic 5 Pro, General Electrics Healthcare, Kranzbuehler/Medizin Systeme Solingen, Germany) sonographic examinations were done between 4 and 8 week, and then transabdominally every 2 weeks until the end of pregnancy. The ultrasonic examination was done weekly between 18 and 20 week. Sheaving of wool or hair 10-20 cm cranial to the udder was done before transabdominal scanning. About 15 ml of ultrasound gel on the transducer was used as a coupling agent for either transrectal or abdominal application to facilitate the transmission of ultrasound waves. The flow angle was maintained as close to 0 degrees as possible. Doppler imaging of each animal took about 20 min.

**Figure 1:**
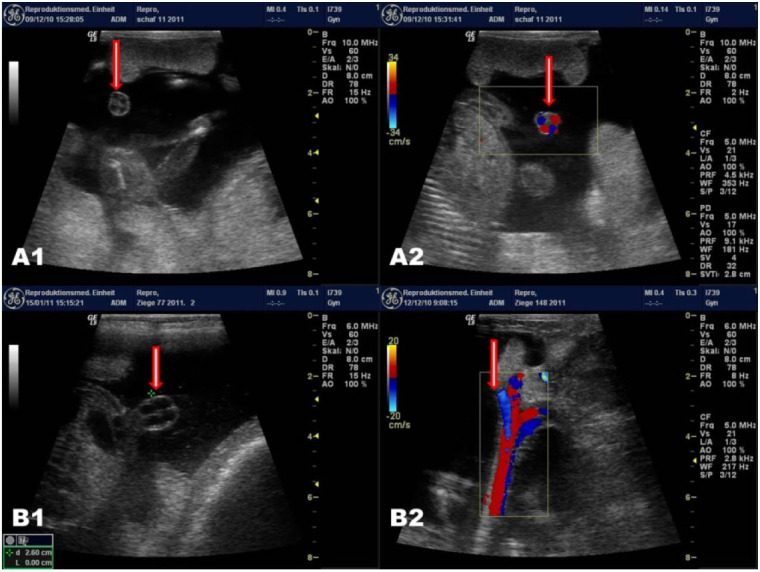
location of umbilical blood vessels (arrow head) in sheep and goat foetuses (A1; B1: B-mode; A2; B2: colour Doppler imaging) respectively.

The location of umbilical blood vessels in small ruminants is dependent on a unique anatomical feature which is the presence of 4 vessels (2 arteries and 2 veins) in the umbilical cord which floats in the amniotic fluid surrounding the foetus ( Fig. 1A , Fig. 1B ). All examinations were done with sheep and goats in the standing position and by the same person (Dr. Mohammed Elmetwally).

The colour Doppler parameters used during this study were: quantitative blood flow volume (BFV), time averaged maximum velocity (TAMV), resistance index (RI), pulsatility index (PI), time average of mean (TAMEAN) and resistance impedance (PS/ED or AB), as well as qualitative descriptions of umbilical blood flow. In case of twin pregnancies, the mean values for both foetuses umbilical blood flow indices were taken during measurements until week 20 of pregnancy in both species.

Doppler velocimetery measurements were taken over 5 to 7 continuous regular waveforms. All measurements of frozen images were recorded automatically by the Doppler ultrasound machine.

### Statistical analysis

The quantitative parameters (BFV, TAMV, PI, RI, TAMEAN and PS/ED) were assessed using normal probability plots and the Kolmogorov-Smirnov test. For the normal distributed parameter, TAMV arithmetic means (x̄) and standard deviations (S.D.) were calculated. Analysis of variance of data from colour Doppler measurements was by one-way analysis of variance (ANOVA) with time points as repeated measurements. Post-hoc multiple pairwise comparisons were done according to the Tukey adjustment of error rate.

The relationship between interval-scaled blood flow parameters was assessed by calculating Pearson’s correlation coefficient r (rho). Analyses were carried out with the statistical software SAS®, version 9.2 (SAS Institute, Cary, NC). For analysis of the linear model, mixed models were used. Significance was defined as P ≤ 0.05.

## Results

**Figure 2:**
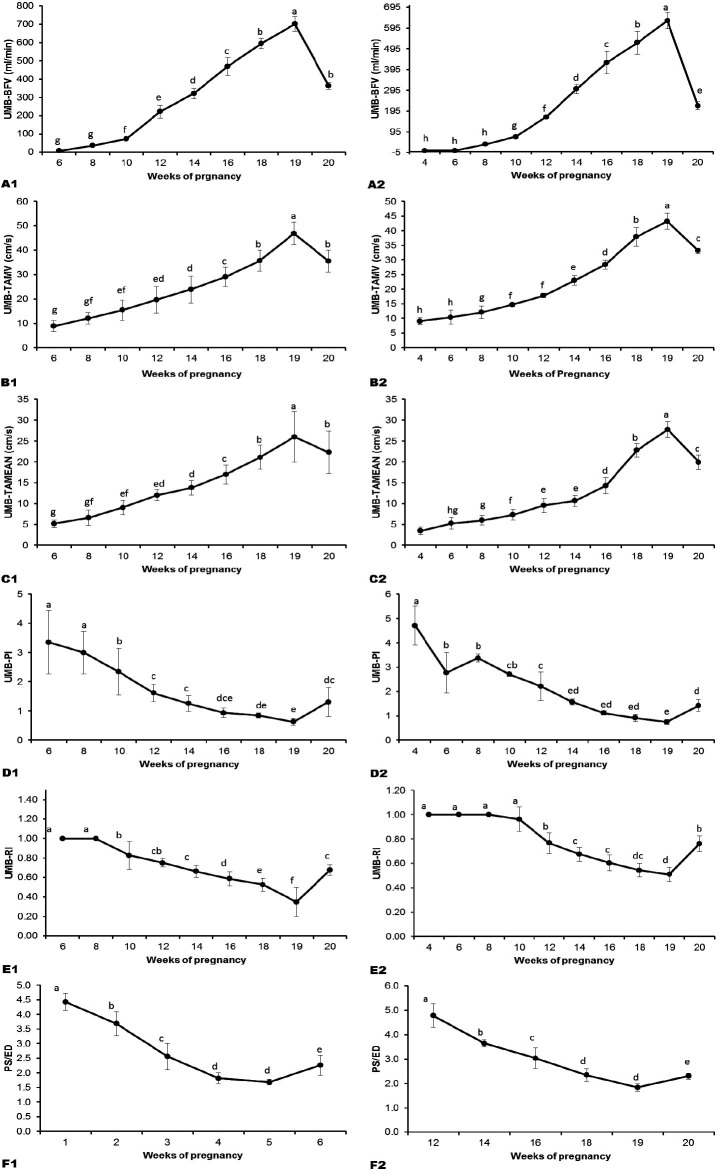
A1. ewes (left)Umbilical artery blood flow volume (BFV) throughout pregnancy. Values are Mean ± SD of 13 pregnant ewes. Means with different superscripts (a,b,c,d,e,f,g) are significantly different (P < 0.05). Figure 2A2. goats (right): Umbilical artery blood flow volume (BFV) throughout pregnancy. Values are Mean ± SD of 13 pregnant goats. Means with different superscripts (a,b,c,d,e,f,g,h) are significantly different (P < 0.05). Figure 2B1. ewes (left)Umbilical artery time averaged maximum velocity (TAMV) throughout pregnancy. Values are Mean ± SD of 13 pregnant ewes. Means with different superscripts (a,b,c,d,e,f,g) are significantly different (P < 0.05). Figure 2B2. goats (right): Umbilical artery time averaged maximum velocity (TAMV) throughout pregnancy. Values are Mean ± SD of 13 pregnant goats. Means with different superscripts (a,b,c,d,e,f,g,h) are significantly different (P < 0.05). Figure 2C1. ewes (left)Umbilical artery Time averaged mean velocity (TAMEAN) throughout pregnancy. Values are Mean ± SD of 13 pregnant ewes. Means with different superscripts (a,b,c,d,e,f,g) are significantly different (P < 0.05). Fig 2C2. goats (right): Umbilical artery Time averaged mean velocity (TAMEAN) throughout pregnancy. Values are Mean ± SD of 13 pregnant goats. Means with different superscripts (a,b,c,d,e,f,g,h) are significantly different (P < 0.05). Figure 2D1. ewes (left) Umbilical artery Pulsitality index (PI) throughout pregnancy. Values are Mean ± SD of 13 pregnant ewes. Means with different superscripts (a,b,c,d,e) are significantly different (P < 0.05). Figure 2D2. goats (right): Umbilical artery Pulsitality index (PI) throughout pregnancy. Values are Mean ± SD of 13 pregnant goats. Means with different superscripts (a,b,c,d,e) are significantly different (P < 0.05). Figure 2E1. ewes (left) Umbilical artery Resistance index (RI) throughout pregnancy. Values are Mean ± SD of 13 pregnant ewes. Means with different superscripts (a,b,c,d) are significantly different (P < 0.05). Figure 2E2. goats (right): Umbilical artery Resistance index (RI) throughout pregnancy. Values are Mean ± SD of 13 pregnant goats. Means with different superscripts (a,b,c,d) are significantly different (P < 0.05). Figure 2F1. ewes (left) Umbilical artery Systolic/Diastolic ratio (S/D) throughout pregnancy. Values are Mean ± SD of 13 pregnant ewes. Means with different superscripts (a,b,c,d,e) are significantly different (P < 0.05). Figure 2F2. goats (right): Umbilical artery Peak Systolic/ End Diastolic ratio (PS/ED) throughout pregnancy. Values are Mean ± SD of 13 pregnant goats. Means with different superscripts (a,b,c,d,e) are significantly different (P < 0.05).

**Figure 3:**
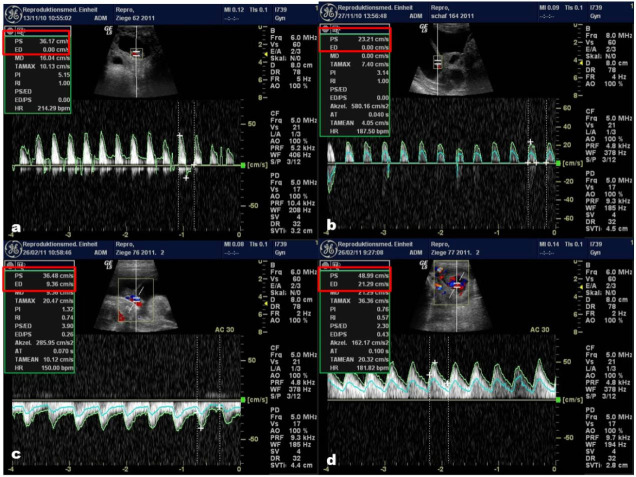
Qualitative characteristics of umbilical arteries blood flow in goats (a: 22 days; ED = 0) and sheep (b: 22 days; ED = 0); c: 16 week of gestation (ED = 9.36; PS= 36.48) and d: 20 week of gestation (ED = 21.29; PS = 48.99)

Parturitions were normal without external assistance for all experimental animals. Twenty five lambs and 41 kids were produced in this study. The assessment of the flow wave in the umbilical arteries included quantitative ( Fig. 2 ) and qualitative assessments ( Fig. 3 ). The results from sheep and goats were similar.

### Quantitative assessment

The umbilical artery BFV increased (P < 0.0001) during pregnancy from 7.27 ± 0.82 ml/min in sheep *vs*. 4.96 ± 0.54 ml/min in goats at 6 week of gestation to 700.51 ± 31.05 ml/min (~100 fold) in sheep *vs*. 665.56 ± 48.22 ml/min (~133 fold) in goats at 19 week and then decreased (P < 0.0001) [NOTE – significantly is redundant to P < 0.0001] to 350.561 ± 72.15 ml/min in sheep *vs*. 215.17 ± 35.06 ml/min in goats at 20 week ( Fig. 2 : A1 and A2, respectively). Similar to BFV, TAMV (8.53 ± 2.05 cm/sec in sheep *vs*. 8.18 ± 1.57 cm/sec in goats at 6 week of gestation) increased (P < 0.0.0001) to 47.13 ± 5.46 cm/sec in sheep *vs*. 43.22 ± 2.41 cm/sec in goats at 19 week and then decreased (P < 0.0001) to 37.88 ± 4.25 cm/sec in sheep *vs*. 33.56 ± 0.87cm/sec in goats at 20 week ( Fig. 2 : B1 and B2, respectively). The TAMEAN (5.23 ± 0.84 cm/sec in sheep *vs* . 4.98 ± 1.25 cm/sec in goats at 6 week of gestation) increased (P < 0.002) to 25.97 ± 4.94 cm/sec in sheep *vs.* 27.48 ± 1.69 cm/sec in goats at 19 week and then decreased (P < 0.0001) to 22.17 ± 4.38 cm/sec in sheep *vs *. 20.84 ± 1.96 cm/sec in goats at 20 week ( Fig. 1 : C1 and C2, respectively) in sheep and goats. Thus, the blood flow indices increased during the first 19 weeks of pregnancy and then decreased until parturition.

In contrast to blood flow velocities and blood flow volume, UMA-PI, UMA-RI and UMA-PS/PD ( Fig. 2 : D1, 2; E1, 2; F1, 2 respectively.) decreased (P < 0.0001) until 19 week of pregnancy after which time they increased. During the first three examinations (6, 10 and 12 weeks) the resistance index value remained equal to 1 due to the absence of EDV ( Fig. 3a, b ).

Positive correlations were determined in both species between UMA-BFV and UMA-TAMV (r = 0.89; P < 0.0001) and UMA-BFV and UMA-TAMEAN (r = 0.86 *vs*. r = 0.87, P < 0.0001). Negative correlations were detected between UMA-BFV and UMA-PI (r = -0.83 *vs* . r = -0.85, P < 0.0001), UMA-BFV and UMA-RI (r = -0.88 *vs*. r = -0.89, P < 0.0001) and UMA-BFV and UMA-AB (r = -0.84 *vs*. r = -0.74, P < 0.0001) in sheep and goats, respectively ( Table 1 , 2 ).

**Table 1:** Pearson's rank correlation coefficients for the relationships between the time averaged maximum velocity (TAMV_UM), Pulsatility index (PI_UM), Resistance index (RI_UM), TAMEAN_UM), Blood flow volume (BFV_UM), Time averaged mean velocity and peak systolic/end diastolic (PS/ED_UM) of umbilical arteries blood flow Doppler parameters (D Parameters) in sheep throughout pregnancy.

	TAMV_UM	PI_UM	RI_UM	BFV_UM	TAMEAN_UM
**TAMV_UM** TAMV_UM	1.00000	-0.75654 <.0001	-0.80265 <.0001	0.89331 <.0001	0.94706 <.0001
**PI_UM** PI_UM	-0.75654 <.0001	1.00000	0.80410 <.0001	-0.83523 <.0001	-0.77866 <.0001
**RI_UM** RI_UM	-0.80265 <.0001	0.80410 <.0001	1.00000	-0.88848 <.0001	-0.80924 <.0001
**BFV_UM** BFV_UM	0.89331 <.0001	-0.83523 <.0001	-0.88848 <.0001	1.00000	0.86656 <.0001
**TAMEAN_UM** TAMEAN_UM	0.94706 <.0001	-0.77866 <.0001	-0.80924 <.0001	0.86656 <.0001	1.00000
**PS/ED_UM** PS/ED_UM	-0.81353 <.0001	0.73662 <.0001	0.67091 <.0001	-0.84234 <.0001	-0.71745 <.0001

Bold parameters are R value and reverse bolding indicate P value.

**Table 2:** Pearson's rank correlation coefficients for the relationships between the Time averaged maximum velocity (TAMV_UM), Pulsatility index (PI_UM), Resistance index (RI_UM), TAMEAN_UM), Blood flow volume (BFV_UM), Time averaged mean velocity and peak systolic/end diastolic (PS/ED_UM) of umbilical arteries blood flow Doppler parameters (D Parameters) in goats throughout pregnancy.

	TAMV_UM	PI_UM	RI_UM	BFV_UM	TAMEAN_UM
**TAMV_UM** TAMV_UM	1.00000	-0.76262 <.0001	-0.75043 <.0001	0.88522 <.0001	0.97599 <.0001
**PI_UM** PI_UM	-0.76262 <.0001	1.00000	0.78970 <.0001	-0.84966 <.0001	-0.78475 <.0001
**RI_UM** RI_UM	-0.75043 <.0001	0.78970 <.0001	1.00000	-0.89219 <.0001	-0.80396 <.0001
**BFV_UM** BFV_UM	0.88522 <.0001	-0.84966 <.0001	-0.89219 <.0001	1.00000	0.87342 <.0001
**TAMEAN_UM** TAMEAN_UM	0.97599 <.0001	-0.78475 <.0001	-0.80396 <.0001	0.87342 <.0001	1.00000
**PS/PD_UM** PS/PD_UM	-0.88030 <.0001	0.75209 <.0001	0.52137 0.0011	-0.74467 <.0001	-0.87634 <.0001

Bold parameters are R value and reverse bolding indicate P value.

### Qualitative assessment

The umbilical artery blood flow in small ruminants is characterized by the absence of end diastolic velocity during the first 3 examinations ( Fig. 3a, b ), but then appeared at 12 week of ultrasonographic colour Doppler scanning ( Fig. 3c, d ). Thereafter, with the progression of pregnancy, both EDV and PSV increased.

## Discussion

Non-invasive colour Doppler ultrasound is a new technique provided with B-mode sonography. It is an efficient and safe diagnostic tool that reflects intrauterine physiological and developmental changes in the fetus during pregnancy.

The results of the present study indicated that blood flow volume showed a consistent change throughout gestation in both sheep and goats. These results indicated an increasing of blood flow to about 700 ml/min and 665 ml/min in sheep and goats foetuses, respectively, near term. These results are similar to those obtained using the Fick principle with urea as a test substance ( Metcalfe and Parer, 1966 ) and electromagnetic flowmeters in fetal lambs (717 ml/min) near term ( Dawes and Mott, 1964 ). Similarly, other studies ( Cooper *et al*., 1949 ; Acheson *et al*., 1956 ) have shown that the umbilical BFV near term in fetal lambs was 500 ml/min. The differences in values from results of the present study are attributed to differences in methodologies. The increase in blood flow volume in the present study is attributed to the simultaneous increase in intrauterine fetal growth ( Gill *et al*., 1981 ) that results from angiogenesis and vasculogenesis/vasodilation ( Link *et al*., 2007 ).

In this study, umbilical BFV decreased after 19 week in sheep and goats foetuses which is in agreement with decreases in BFVs recorded for equine ( Bollwein *et al*., 2004 ) and human foetuses ( Gill *et al*., 1981 ; McGladdery *et al*., 1992 ; Link *et al*., 2007 ). In human foetuses, umbilical blood flow decreased after 37 week of gestation. The reduction in umbilical blood flow may be primary or secondary to changes in the uteroplacental circulation ( Kurjak and Rajhvajn, 1982 ) as well as a result of fetal hypoxia ( Link *et al*., 2007 ). Also, blood flow changes to meet metabolic demand, so a slower growing fetus may require less blood flow for delivery of nutrients and gases.

The umbilical TAMV and TAMEAN values increased during pregnancy, which can be attributed to the increase in the diameter of the umbilical blood vessels in addition to increased fetal [the rate of fetal growth is decreasing] and placental weights [placental weights in sheep change little after day 75 to 90 of gestation] with advancing of gestation. Furthermore, Stuart *et al*. (1980) attributed increases in TAMV to the decrease in vascular impedance and increase in vascular perfusion to the foetus. The reduced values of these indices after 19 week of gestation may be attributed to the decrease in fetal blood pH close to term as a result of limited fetal growth and decreased blood flow volume during this period.

In our study, the umbilical resistance and pulsatility indices of the umbilical vessels, as well as resistance impedance decreased steadily during pregnancy until 19 week and then increased until parturition. These results are in agreement with findings of Erskine and Ritche (1985) who reported reductions in those indices during pregnancies with normal development of human foetuses. As well, decreases in the umbilical resistance index were recorded for equine foetuses until the mid-gestation and then increases during the few weeks prior to foaling ( Bollwein *et al*., 2004 ). Similarly, in Saanen goats, umbilical PI and RI by days 85 and 130 of gestation were decreased and this may be attributed to changes in fetal nutrient requirements ( Serin *et al*., 2010 ). Moreover, in our study, we assessed the umbilical resistance impedance index non-invasively in small ruminants (AB or PS/ED ratio) which is the most important colour Doppler index that indicates the variation in end diastolic velocity especially during the second and third trimester ( Erskine and Ritchie, 1985 ) reflecting normal intrauterine fetal growth and fetoplacental development in late pregnancy.

The increase in umbilical PI and RI after 19 week of gestation may be caused by a decrease in fetal growth rate which subsequently leads to reduced blood flow and decrease in oxygen tension in fetal blood. Moreover,

Bollwein *et al*. (2004) attributed the increase in umbilical RI prior to foaling to a decrease in fetal blood perfusion.

In the current study, the EDV was absent at 4 and 8 wks of gestation ( Fig. 3 ), while the first appearance was at 12 week of gestation in both ewes and goats. Our results are similar to those in the human foetus in which the EDV was detected between 12 and 14 weeks before being continuous after 14 week of gestation in the same foetus ( Coppens *et al*., 1996 ). We believe that the appearance of the EDV was associated with the regularity of fetal cardiac cycles and decreased fetal heart rate ( Elmetwally, 2012 ). Furthermore, the appearance of EDV has been attributed to a decrease in umbilical artery PI ( Coppens *et al*., 1996 ).

In conclusion, non-invasive Colour Doppler sonography can be used safely to follow the hemodynamics changes in umbilical blood flow in pregnant sheep and goats. Additionally, results of the present study strongly suggest that this technique can be used to evaluate intrauterine fetal growth retardation in clinical studies.
